# Transcutaneous compression suture with iodine gauze for wound closure in revision surgery for therapy-resistant periprosthetic shoulder infections

**DOI:** 10.1016/j.jseint.2023.08.007

**Published:** 2023-09-09

**Authors:** Florian Grubhofer, Evan O’Donnell, Lukas Ernstbrunner, Ilker Uçkay, Karl Wieser

**Affiliations:** a Department of Orthopedics, Balgrist University Hospital, University of Zürich, Zürich, Switzerland; b Department of Orthopaedics, Massachusetts General Hospital, Harvard Medical School, Boston, MA, USA; c Department of Orthopaedic Surgery, Royal Melbourne Hospital, Parkville, VIC, Australia; d Department of Biomedical Engineering, University of Melbourne, Parkville, VIC, Australia

**Keywords:** Transcutaneous compression suture, Fragile wound conditions, Periprosthetic shoulder infection, Iodine gauze compression suture, Revision shoulder surgery, Persistent drainage

## Abstract

**Background:**

To successfully treat a periprosthetic infection, successful bacteria eradication and successful wound closure are mandatory. Despite adequate surgical débridement in the deep, persistence of wound drainage and subsequent persistence of periprosthetic infection may occur, especially in patients with compromised soft tissue conditions. This study presents a transcutaneous compression suture technique with iodine gauze that was used in patients with persistent wound secretion in therapy-resistant periprosthetic shoulder infections in order to achieve successful infection control and wound healing.

**Methods:**

All patients with persistent periprosthetic or implant-associated shoulder joint infections despite correct previous surgical and antibiotic therapy attempts were included in the study. In all patients, in addition to repeat deep surgical débridement, a transcutaneous “iodine-gauze-compression-suture” was performed with postoperative antibiotic therapy. The primary endpoint was infection control; the secondary endpoint was wound healing rate; and the third endpoint was complication rate after index surgery.

**Results:**

Ten consecutive patients with a mean age of 74 (±7.6) years and a mean follow-up of 14 (±2) months were included. All ten patients showed infection control and successful wound healing, with no need for further revision surgery. In 8 out of 10 patients, the wound healing was fast and completely uncomplicated. Two out of 10 patients showed delayed wound healing with fibrin coatings for 3 and 4 weeks, respectively. No additional intervention was necessary in both patients.

**Conclusions:**

Transcutaneous iodine gauze compression sutures were used to achieve successful infection control without additional revision surgery in 10 out of 10 patients who previously underwent surgery with failed infection control. This wound closure technique is a reliable adjunctive therapy method in the treatment of implant-associated infections of the shoulder in patients with fragile wound conditions.

In patients with periprosthetic or implant-associated infections, the focus is usually on the eradication of the deep joint infection, which may involve radical débridement of infected bone and soft tissue, removal of the implant, implantation of an antibiotic-loaded cement spacer, and perioperative antibiotic therapy.^6^^,^^10^^,^^15^ Despite adequate surgical treatment of the deep infection and appropriate antibiotic therapy, persistence of the infection may occasionally occur.^13^ Particularly in patients with numerous comorbidities and prior surgeries, the skin and subcutaneous tissue may be compromised, which is a substantial risk for postoperative wound healing complications, infection persistence or even a potential source for a new infection with a different pathogen.^3^ Accordingly, it is important that in the context of the surgical treatment, in addition to radical deep débridement, the soft tissues are optimally conditioned during wound closure to allow for successful wound closure. Without successful wound closure, infection control is not possible. Due to fragile skin and subcutaneous tissue, the sutures used for wound closure are often insufficient to provide the necessary compression needed to reduce the subcutaneous dead space, which is a risk factor for hematoma or seroma formation.^16^^,^^18^ This can subsequently lead to wound drainage with the risk of persistent infection. Various wound closure techniques have been developed to improve postoperative wound healing in such challenging soft tissue conditions. For particularly thin skin, such as in elderly patients due to loss of collagen content, techniques using strips, skin adhesives, the combination of strips and sutures, or the use of a skin-reinforcing gauze bandage in combination with sutures have been described.^7^^,^^8^^,^^11^^,^^12^ These closure techniques only provide closure of the superficial portions of the wound.^14^ Negative pressure wound closure systems can increase pressure on the deep portions of the wound to counteract the undesirable accumulation of fluid.^1^ However, these systems are more expensive, and the tube system with the portable collector system and the portable vacuum generation system that creates disturbing noises may be less practical for patients compared to a wound suture. This study describes a wound closure technique based on 10 consecutive patients that aims to reduce the subcutaneous dead space while protecting the superficial skin layer in order to reliably achieve successful wound healing and infection control.

## Methods

### Patients

After obtaining institutional review board approval from the local ethics committee, a retrospective analysis of all patients treated with an iodine gauze compression suture (IGCS) for an uncontrolled periprosthetic shoulder joint infection (PSJI) between January 2017 and December 2021 was performed. All patients, who met International Consensus Meeting (ICM) shoulder group^9^ for a PSJI with persistence of PSJI despite prosthesis/implant removal, cement spacer implantation or 1-stage revision who were treated with a débridement, cement spacer exchange, or implantation in combination with an IGCS were included in the study. The definition of periprosthetic shoulder infection was based on the definition of the ICM shoulder group (ICM shoulder group).^9^ The minimum follow-up was 12 months.

### Compression suture technique

**Figure 1:**
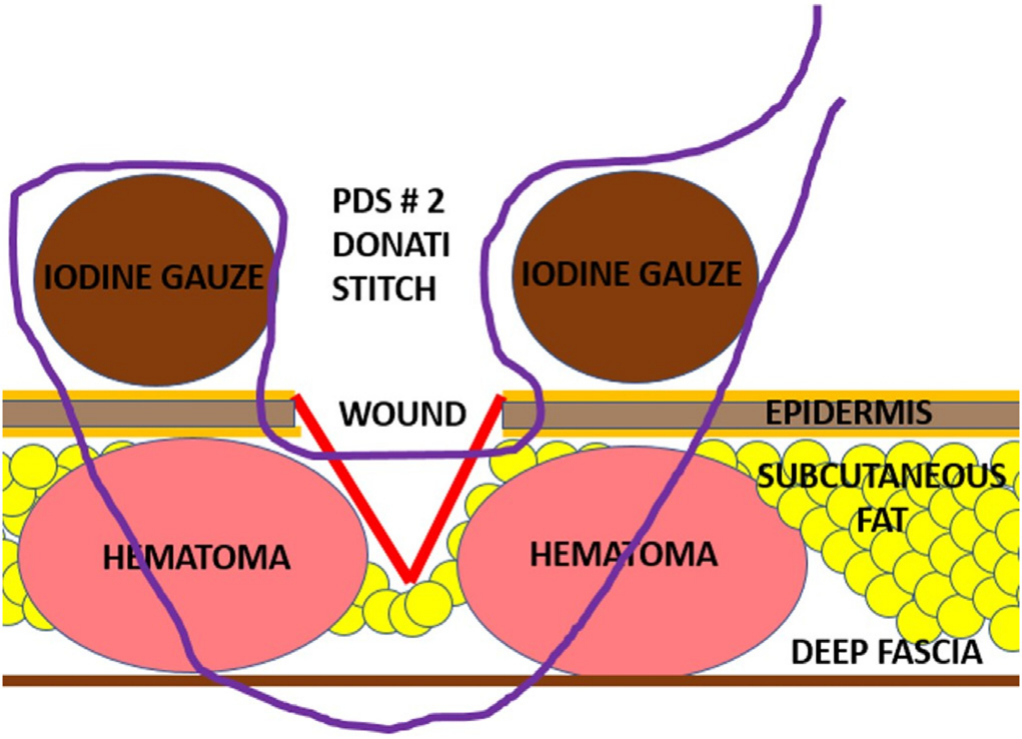
Animation of the compression suture stich with the Nr. 2 PDS suture (purple line) and the placement of the iodine gauze on the skin (brown circles). The figure demonstrates the course of the PDS suture course starting with the first deep stich through the deep fascia followed by the second superficial stich that brings together the wound edges of the skin. *PDS*, polydioxanone suture.

**Figure 2:**
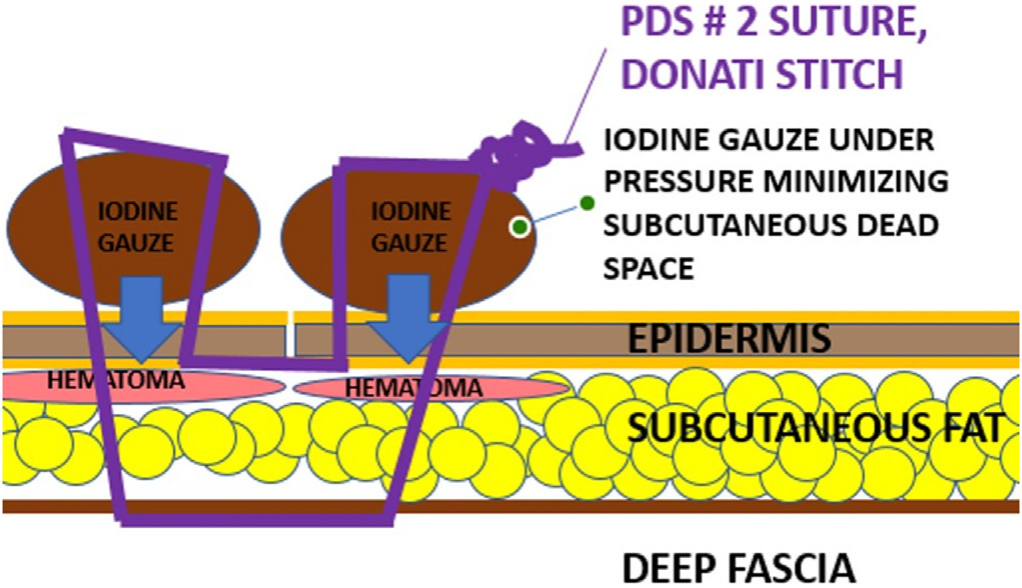
Animation of the compression suture after knotting the PDS suture. By pulling and knotting the PDS suture, a compression force is developed between the deep fascia and the superficial iodine gauze, which minimize the subcutaneous dead space and the subcutaneous hematoma (red circle). *PDS*, polydioxanone suture.

**Figure 3:**
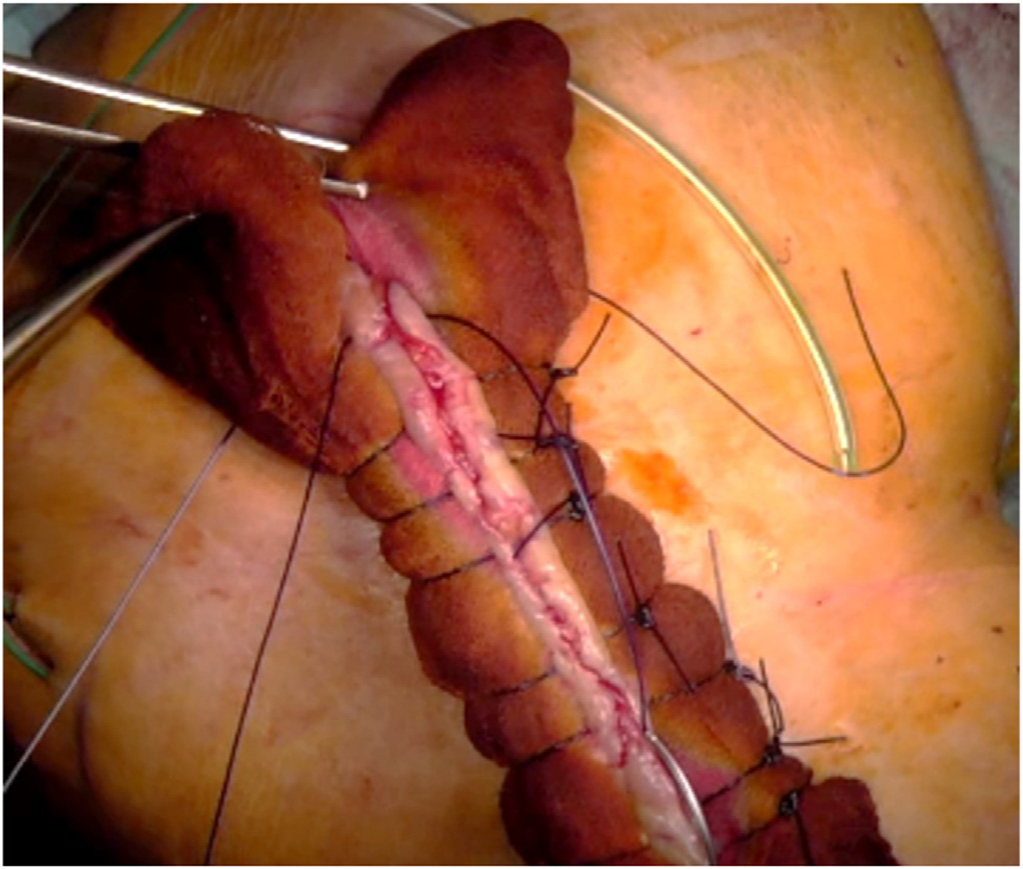
Intraoperative picture of an iodine gauze compression suture in a shoulder wound of a 79-year-old male patient with 9 previous surgeries due to a periprosthetic shoulder joint infection. The compression suture was applied after deep surgical débridement and an exchange of the spacer. After application of the suture the skin seems vital without any signs of strangulation or underperfusion.

In addition to the repeated deep radical wound débridement with intraoperative sampling (5 tissue cultures and 1 histology) taken in every case, if necessary, cement spacer insertion or spacer exchange, a so-called IGCS technique was additionally used for wound closure. The surgeries were all performed by the same surgeon (F.G). For the compression suture technique, an iodine-soaked gauze was placed on each side of the wound and then secured onto the skin with Donati stitches using a 2 polydioxanone suture (PDS) suture. The gauze was soaked in a nonalcoholic iodine standard solution (Betadine; Mundipharma AG, Basel Switzerland; concentration: 11mg povidonum iodinatum, per ml). The first lateral stitches of the Donati stitch were deep stitches through the skin, subcutaneous fat layer, and deep fascial layer. The medial stitches were superficially placed to bring the wound edges of the skin close together. By pulling and knotting the PDS suture, pressure was created between the superficial gauze and the deep fascia, which reduced the dead space volume in the subcutaneous fat layer. The interposed superficial gauze is to protect the skin, which without the gauze could be strangulated and damaged by the powerful knotting that is needed for the compression suture. (Figure 1, Figure 2, Figure 3 and Video).

The compression suture was left in place for 14 days. All patients were immobilized in a sling for 6 weeks. The gauze of the compression suture was soaked in iodine daily to avoid contamination of the gauze. Wound care was performed by either an outpatient wound care service or the nursing staff at a rehabilitation facility. All patients were treated with intravenous antibiotic drugs between 3 and 7 days until the first results of the tissue cultures were obtained followed by oral antibiotic therapy for 6 to 12 weeks depending on the intraoperative cultures. The antibiotic therapy was determined by the Department of Infectious Diseases at our hospital.

### Measurements

The primary outcome parameter was the infection control rate after the index surgery. Infection control was defined as successful wound closure in the absence of wound drainage and absence of elevated C-reactive protein, erythrocyte sedimentation rate, or white blood count. The primary endpoint parameter was assessed by the operating surgeon (F.G.) together with an infectious disease specialist (I.U.) during the 6- and 12-week postoperative visits. All patients and their infection status (infection controlled, eradicated, etc.) were discussed at weekly infection disease rounds between the orthopedic and infection disease department. The reoperation rate, including all surgeries performed for wound revision or infection treatment was the second outcome parameter assessed during the 6- and 12-month postoperative visits. Postoperative complications were assessed using the Clavien–Dindo classification score and recorded as the third outcome parameter^5^ and was assessed during the 2, 6, and 12-week postoperative visits. The complication system described by Clavien–Dindo is a standardized complication system especially developed for wound healing problems in surgery.^1^ The Clavien–Dindo classification is the most common cited publication in surgery in history.^3^ The wound healing process was photo documented in all patients preoperatively, after application of the compression suture, after removal of the compression suture, and after completed wound healing.

## Results

### Patients

**Figure 4:**
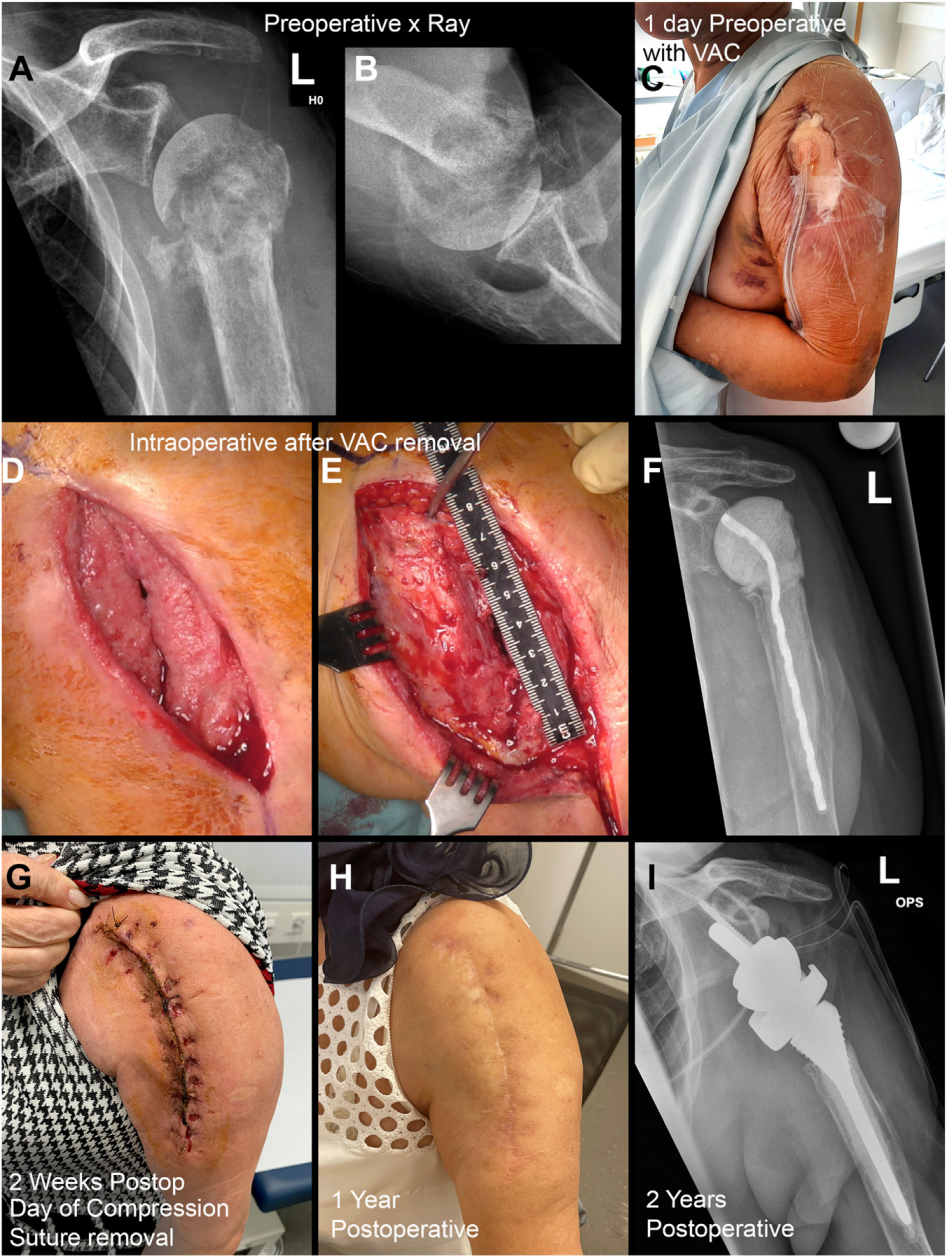
Clinical course of a 60-year-old female patient with an implant-associated *Staphylococcus aureus* infection of her left shoulder. She had a total of 8 prior surgeries (including osteosynthesis, removal of the plate, multiple débridement, negative pressure wound therapy, implant removal, removal of the humeral head, and implantation of an antibiotic cement spacer and intravenous antibiotic therapy) in which no infection control was achieved. After spacer exchange, débridement, and application of an iodine gauze compression suture and the wound was dry after 5 days, and the wound was closed after removal of the compression suture 2 weeks postoperatively. The fibrin deposits healed without any further intervention. After 1 year, the patient underwent a reverse total shoulder arthroplasty. The 5 intraoperative cultures and the histology were all negative. 1 year after RTSA patient is pain free and reports a subjective shoulder value of 80%. a-b) ap x-ray of left shoulder after 7 previous surgeries and before spacer implantation + compression suture, c) picture of the wound with negative pressure wound therapy before spacer implantation + compression suture, d) intraoperative picture after removing the negative pressure wound therapy, and e) after débridement of the wound, f) AP x-ray of the shoulder after insertion of the spacer + compression suture, g) 2 weeks postoperative after removing of the compression suture, h) 1 year after compression suture, and i) ap x-ray of the left shoulder after removal of the spacer and implantation of RTSA 1 year after spacer insertion in combination with compression suture. *RTSA*, reverse total shoulder arthroplasty.

A total of 10 consecutive patients, who all fulfilled the ICM criteria for PSJI with an average age of 74 years (SD 7.6 years) with a mean follow-up of 14 (±2) months (minimum follow-up of 12 months) were included in the study. Of the 4 women and 6 men, 8 patients (80%) had undergone previous surgeries at external hospitals and were referred to our clinic for further treatment of the therapy-resistant periprosthetic or implant-associated shoulder joint infection. The number of previous surgeries performed for infection control or wound closure varied between 1 and 3 in eight patients and 8 and 9 in two patients – see Fig. 4. In all 10 patients, the infected shoulder arthroplasties or implants were removed, and a radical débridement was performed with the achievement of infection control. All patients had antibiotic therapy after the failed prior surgery.

### The prior surgeries that were performed without achievement of infection control were

Removal of the arthroplasty, débridement, and implantation of an antibiotic-loaded cement spacer in the setting of a two-stage approach in 6 cases; removal of the arthroplasty, débridement, and reimplantation of a new implant in the setting of a one-stage approach in 2 cases; and removal of the plate, débridement, cement spacer implantation, and negative pressure wound therapy in two cases.

The IGCS was combined in 5 cases with an antibiotic spacer exchange, and in another 5 cases, the compression suture was combined with the removal of the implant or the humeral head and implantation of a new antibiotic-loaded cement spacer.

Most patients were severely ill with an American Society of Anesthesiologists score of III (n = 4) or IV (n = 5) and accordingly had significantly reduced general health status.^2^ One patient had an American Society of Anesthesiologists II score.

The pathogens were *Staphylococcus aureus* in 5 cases (2x MRSA), *Corynebacterium acnes* in 2 cases, *Staphylococcus epidermidis* in 1 case, *Streptococcus mitis/oralis* in 1 case, and *Pseudomonas aeruginosa* in 1 case.

### First-, second- and third-outcome measurement

Infection control: all wounds were dry at postoperative day 3 and all patients showed successful wound healing after removal of the compression suture with no signs of infection. In all patients, the infection laboratory values were normal at the 6 and 12-week follow-up, with no evidence of a persistent infection. The infection control rate was therefore 100%.

Revision surgery: no patient needed a revision surgery to achieve infection control. After acheiving infection control, 7 patient underwent a reimplantation of a shoulder arthroplasty (3x RTSA, 4 bipolar shoulder hemiarthroplasty) to improve shoulder function or shoulder pain. In all 7 patients, the tissue cultures and histology (minimum 5 tissue cultures and 1 histology) during the reimplantation were negative, so infection eradication was proven in these cases. The three patients, in whom the cement spacer was left in place had no or little pain and were not limited in their quality of life; therefore, reimplantation of a shoulder prosthesis was not indicated. In these 3 patients, the cultures were negative when the spacer was inserted and the compression suture was applied, additionally, the last CRP and leucocyte levels were within normal limits, so that infection eradication is very likely – Table I.

**Table I:** List of included patients with number of previous infection-related surgeries, infection-causing therapy-resistant pathogen, intraoperative sampling results, pre and postoperative laboratory infection markers.

	Number of previous infection related surgeries	Pathogen	Pathogen at last revision and compression suture application	CRP pre- vs postop (mg/L)	Leucocyte pre- vs. postop (G/l)	Blood sedimentation pre vs. postop (mm/h)
Patient 1	2 (1x implant removal with spacer implantation, 1x exchange of spacer)	*Staph. epidermidis*	negative	76-6	13.4-5.8	45-n/a
Patient 2	1 (1x implant removal, spacer implantation)	*Staph. aureus*	negative	121	6.3-8.2	98-18
Patient 3	8 (1x plate removal + débridement, 6x débridement + negative pressure wound therapy, 1x spacer implantation)	*Staph. aureus* (MRSA)	negative	100-7	8.6-6.6	30-12
Patient 4	2 (1x wash out and exchange of mobile components, 1x implant removal and spacer implantation)	Streptococcus Mitis/Oralis	4/5 positive with Streptococcus mitis/oralis	101-8	8.3-5,9	27-2
Patient 5	3 (1x implant removal + spacer, Débridement, Reimplantation RTSA, implant removal and spacer)	*Staph. aureus* (MRSA)	negative	20-4	8.6-5.9	48-n/a
Patient 6	1 (implant removal and spacer implantation)	*C.acnes*	negative	23-1.8	9.5-4.1	40-17
Patient 7	9 (1x implant removal + débridement, 8x débridement	*Staph. aureus*	negative	210-4	10.9-7.6	n/a
Patient 8	3 (1x removal of mobile components, 1x implant removal + spacer, 1x superficial wound revision)	*C. acnes*	negative	29-3	6.5-4.9	53-11
Patient 9	2 (1x implant removal + spacer, 1x spacer exchange)	*Staph. aureus*	negative	38-2	9.7-5	38-n/a
Patient 10	2 (1ximplant removal + spacer, 1xspacer exchange)	Pseudomonas Aeroginosa	negative	95-4	10.6-5.7	n/a

*C. acnes*, corynebacterium acnes; *CRP*, C reactive protein; *MRSA*, methicillin-resistant staphylococcus aureus.

Complications: In two patients, fibrin deposits were observed for 3 and 4 weeks postoperatively, respectively. These fibrin deposits were managed with dry dressings, and both patients achieved spontaneous wound healing without requiring additional interventions. However, these two fibrin deposits were classified as wound healing problems and graded as Clavien–Dindo grade II complications, as additional wound check appointments were required.

## Discussion

In addition to adequate surgical and medical treatment of deep implant-associated joint infections, adequate treatment of superficial soft tissues including wound closure is an essential component of successful infection treatment.

In these 10 consecutively treated patients, prior surgeries in which radical débridement and removal of the arthroplasty/implant were performed did not achieve infection control. This consecutive patient cohort demonstrates that adequate “deep” surgical débridement alone including débridement with implant removal combined with spacer implantation or one-stage exchange, is sometimes not sufficient to achieve successful infection control. In the surgical treatment of PJI, which affects typically elderly patients with multiple comorbidities, it is essential to optimize soft tissue management. The compression suture seemed to be safe and helpful in this patient series to achieve wound closure and infection control. The hypothesis behind the compression suture technique is that the developed compression between the incorporated gauze and the deep fascial layer decreases dead space volume in the subcutaneous fat layer and therefore reduces the risk for hematoma or seroma formation, which are risk factors for persistent wound drainage and wound infections.^4^^,^^18^ We assume that the equal pressure distribution over the interposed gauze and the simultaneous reduction of the strangulation effect of conventional knotting techniques represent the main effects of favorable wound conditioning. In our opinion, the use of iodine as a disinfectant plays a rather minor role. The disinfectant is used primarily for continuous disinfection of the inserted gauze, which remains on the skin for two weeks, and secondarily to maintain the uniform brown color of the gauze dressing. If white gauze is used, a bloody red discoloration may occur during suture application when the gauze touches the bloody wound edge, which may be subjectively disturbing to patients who must wear a bloodstained dressing on their skin for two weeks. Due to the iodine-induced brown coloration of the gauze, the occasional blood discoloration was not visible. Initial concerns about dermal damage or skin irritation due to local iodine application for several weeks did not occur. In two patients, small fibrin deposits were visible at the 2-week follow-up, which healed without further intervention. The wound edges and the skin beneath the compression suture appeared after removal more vital compared to preoperative conditions, so that it can be assumed that the immobilization of the skin and soft tissue generated by the very stable compression suture has a positive effect on the rehabilitation of the skin. In addition, there was concern that the pressure generated by tightening the knots might be too high for the skin and could cause skin necrosis. However, this concern was not confirmed in the 10 cases, even though 2 patients had fibrin deposits of less than 10 mm and healed without any further intervention after 3 and 4 weeks. Nevertheless, even in the two patients with the fibrin deposits, the skin condition improved in all 10 patients after application of the compression suture compared to the preoperative status. It is possible that the interposed gauze reduces the strangulating effect of the knots on the skin, which usually occurs with conventional knot techniques, and thereby reduces compromise of blood supply. However, this hypothesis would need to be confirmed by measuring the skin oxygen partial pressure, which was not performed in this study.^17^

The radical deep débridement of the infection with removal of all infected implants and infected bone fragments is a prerequisite for successful infection control, and the compression suture should only be considered an adjuvant therapy to optimize the soft tissue situation. It cannot in any way replace deep infection eradication.

After the compression suture was successfully and without complications used in the shoulder area, it was also used in other joint areas (knee, foot and ankle, and elbow) with comparable successful wound healing rates.

Limitations: This is a retrospective analysis of a small patient series without a control group, in which a conventional suture technique would have been applied. However, it should be noted that in all patients, prior surgical interventions, including débridement and implant removal, failed to achieve infection control. In this situation, generating a control group that would theoretically undergo the same surgical intervention with the same wound closure that was unsuccessful in the previous surgery would not be justifiable. The definition of infection eradication in this study can be debated. Objectively, only the blood values of ESR, CRP, and white blood count were evaluated, which per se are not reliable markers. The laboratory values alone are only part of the evaluation. An essential part is the clinical evaluation of the wound by the orthopedic and infection disease department as well as the absence of pain. In all patients, the wound was dry and without evidence of persistent infection (no redness, swelling, secretion, etc.) after 14 days. In addition, all patients were pain-free. Laboratory values were also within the normal range in almost all patients. Based on these clinical and laboratory results, the infections were classified as eradicated both from the orthopedic side and from the infection disease side. In our opinion, invasive diagnostic procedures such as puncture,biopsy, or even arthroscopic sampling are scientifically interesting but clinically not indicated. For this reason, these procedures were not indicated in asymptomatic patients. To increase the size of the study, other centers that treat such severely ill patients with therapy-resistant implant-associated joint infections could have been included.

## Conclusion

The compression suture described in this study contributed to successful infection control in all 10 predominantly severely ill patients. None of the affected patients required further infection control surgery after the application of the compression suture. The described wound closure technique is safe and has no significant complications.

## Disclaimers

Funding: No funding was disclosed by the authors.

Conflicts of interest: Karl Wieser receives payments as a consultant for Zimmer Biomet, Arthrex, Inc. and Karl Storz SE & Co KG not related to this work. The other authors, their immediate families, and any research foundation with which they are affiliated have not received any financial payments or other benefits from any commercial entity related to the subject of this article.

## Supplementary data

Video Intraoperative video of the wound and the wound closure with an iodine gauze compression suture in a shoulder wound of a 79-year-old male patient with 8 previous surgeries due to a periprosthetic shoulder joint infection. In the first 5 seconds, the original wound is seen. The compression suture was applied after deep surgical débridement and an exchange of the antibiotic loaded cement spacer. The patient had successful wound healing two weeks after the surgery. At the last follow-up visit 13 months after the surgery patient had no revision surgery in the meantime and he is pain free.

## References

[1] Agarwal P., Kukrele R., Sharma D. (2019). Vacuum assisted closure (VAC)/negative pressure wound therapy (NPWT) for difficult wounds: a review. J Clin Orthop Trauma.

[2] American Society of Anesthesiologists Task Force on Preanesthesia Evaluation (2002). Practice advisory for preanesthesia evaluation: a report by the American society of anesthesiologists task force on preanesthesia evaluation. Anesthesiology.

[3] Carroll K., Dowsey M., Choong P., Peel T. (2014). Risk factors for superficial wound complications in hip and knee arthroplasty. Clin Microbiol Infect.

[4] Cheung E.V., Sperling J.W., Cofield R.H. (2008). Infection associated with hematoma formation after shoulder arthroplasty. Clin Orthop Relat Res.

[5] Clavien P.A., Barkun J., de Oliveira M.L., Vauthey J.N., Dindo D., Schulick R.D. (2009). The Clavien-Dindo classification of surgical complications: five-year experience. Ann Surg.

[6] Cooper M.E., Trivedi N.N., Sivasundaram L., Karns M.R., Voos J.E., Gillespie R.J. (2019). Diagnosis and management of periprosthetic joint infection after shoulder arthroplasty. JBJS Rev.

[7] Davis M., Nakhdjevani A., Lidder S. (2011). Suture/Steri-Strip combination for the management of lacerations in thin-skinned individuals. J Emerg Med.

[8] Foster R.S., Chan J. (2011). The Fixomull skin support method for wound closure in patients with fragile skin. Australas J Dermatol.

[9] Garrigues G.E., Zmistowski B., Cooper A.M., Green A., Group ICMS (2019). Proceedings from the 2018 International Consensus Meeting on orthopedic infections: the definition of periprosthetic shoulder infection. J Shoulder Elbow Surg.

[10] Grubhofer F., Imam M.A., Wieser K., Achermann Y., Meyer D.C., Gerber C. (2018). Staged revision with antibiotic spacers for shoulder prosthetic joint infections yields high infection control. Clin Orthop Relat Res.

[11] Joyce K., Potter S. (2020). A novel skin closure technique for the management of lacerations in thin-skinned individuals. Cureus.

[12] Kitcat M., Abdaal A., Durrani A. (2017). Preventing the cheese-wire effect by combining steri-strips and sutures for the management of lacerations in thin-skinned individuals. J Plast Reconstr Aesthet Surg.

[13] Mortazavi S.M., Vegari D., Ho A., Zmistowski B., Parvizi J. (2011). Two-stage exchange arthroplasty for infected total knee arthroplasty: predictors of failure. Clin Orthop Relat Res.

[14] Pacifico M.D., Teixeira R.P., Ritz M. (2009). Suturing of fragile skin. J Plast Reconstr Aesthet Surg.

[15] Parvizi J., Adeli B., Zmistowski B., Restrepo C., Greenwald A.S. (2012). Management of periprosthetic joint infection: the current knowledge: AAOS exhibit selection. J Bone Joint Surg Am.

[16] Patel V.P., Walsh M., Sehgal B., Preston C., DeWal H., Di Cesare P.E. (2007). Factors associated with prolonged wound drainage after primary total hip and knee arthroplasty. J Bone Joint Surg Am.

[17] Sen C.K. (2009). Wound healing essentials: let there be oxygen. Wound Repair Regen.

[18] Shahi A., Boe R., Bullock M., Hoedt C., Fayyad A., Miller L. (2019). The risk factors and an evidence-based protocol for the management of persistent wound drainage after total hip and knee arthroplasty. Arthroplast Today.

